# The Protective Effect of Cordymin, a Peptide Purified from the Medicinal Mushroom *Cordyceps sinensis*, on Diabetic Osteopenia in Alloxan-Induced Diabetic Rats

**DOI:** 10.1155/2013/985636

**Published:** 2013-09-22

**Authors:** Wei Qi, Yang Zhang, Ya-bo Yan, Wei Lei, Zi-xiang Wu, Ning Liu, Shuai Liu, Lei Shi, Yong Fan

**Affiliations:** ^1^Department of Orthopedics, Xi Jing Hospital, The Fourth Military Medical University, Xi'an 710032, China; ^2^The Surgery Department of 520th Hospital of PLA, Mian Yang 621000, China

## Abstract

The aim of this study was to investigate the protective effect of cordymin on diabetic osteopenia in alloxan-induced diabetic rats and the possible mechanisms involved. The diabetic rats received daily intraperitoneal injection with cordymin (20, 50, and 100 mg/kg/day) for 5 weeks. Cordymin could restore the circulating blood glucose, glycosylated hemoglobin (HbA1c), serum alkaline phosphatase (ALP), tartrate resistant acid phosphatase (TRAP), and insulin levels in a dose-dependent manner. Also, the treatment of diabetic rats with cordymin could partially reverse the **β** cells death and decrease the total antioxidant status (TAOS) in the diabetic rats. The results may directly and indirectly account for the possible mechanism of the beneficial effect of cordymin on diabetic osteopenia, which was confirmed with the increased bone mineral content (BMC) and bone mineral density (BMD) in diabetic rats (*P* < 0.05). All those findings indicate that cordymin may play a protective role in diabetic osteoporosis.

## 1. Introduction

**Figure 1:**
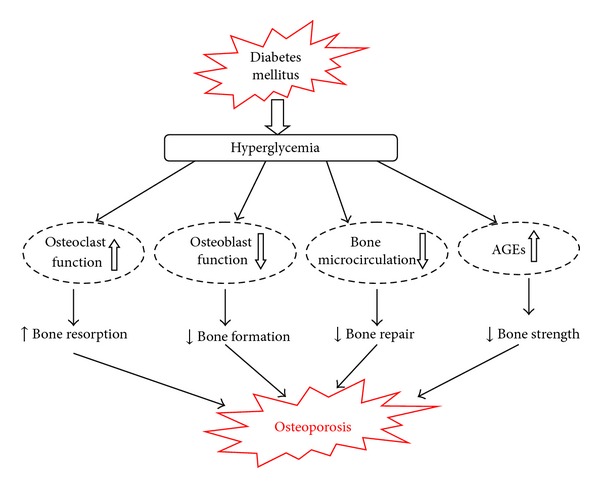
Possible mechanisms through which diabetic conditions increase the risk of osteoporotic fractures (AGEs: advanced glycation end products).

An increasing number of diabetic patients are affected by chronic complications, such as cardiovascular disease, kidney disease, diabetes erectile dysfunction, and diabetic osteoporosis [1–4]. The association between diabetes and decreased BMD has been confirmed in adults (Figure 1), and BMD appears to be decreased in both the spine and hip in diabetic patients [5, 6]. These associations may be related to increased risk of fractures in individuals with diabetes, increased risk of diabetes mellitus in individuals with osteopenia, or both. Growing evidence from clinical studies indicates that osteopenia always leads to an increased incidence of bone fracture and a delay in healing of bone fractures and affects the quality of life in diabetic patients [7]. Therefore, searching for effective drugs which can control the development of diabetic osteopenia is of great significance for patients with diabetic osteopenia.

*Cordyceps sinensis* (CS) has been used as a tonic for longevity, endurance, and vitality for thousands of years by the Chinese. Many studies have shown that CS regulates insulin sensitivity [8] and decreases plasma cholesterol levels [9]. The effect of* Cordyceps sinensis* on osteoporosis had been studied in our former paper [10]. So we herein hypothesize that *Cordyceps sinensis* will be beneficial in preventing osteopenia in diabetes and influencing the longer-term course of glycemic control. We undertook the present study to ascertain if cordymin, a peptide purified from the medicinal mushroom *Cordyceps sinensis*, could be accounted for the putative beneficial effect of CS on diabetic osteopenia in diabetic rats.

## 2. Material and Methods

### 2.1. Preparation of Cordymin

*Cordyceps sinensis* was collected from Qing Hai, China. Cordymin was prepared by the way introduced by Wong et al. [11]. Briefly, dried fruiting bodies of *Cordyceps sinensis* (100 g) were homogenized in liquid nitrogen with a pestle, extracted in distilled water and centrifuged. To the resulting supernatant, ammonium acetate buffer (pH 4.5) was added until a final concentration of 20 mM was attained. The sample was loaded on an SP-Sepharose column. The adsorbed fraction was eluted with 1 M NaCl in 20 mM ammonium acetate buffer (pH 4.5), then dialyzed against distilled water, and lyophilized. Then it was dissolved in 20 mM NH_4_OAc buffer (pH 4.5) and applied on a Mono S column and eluted with the same buffer. The fraction containing cordymin was concentrated and then purified on a Superdex 75 column in the same buffer. The single peak eluted constituted purified peptide designated as cordymin.

The molecular mass determination of cordymin was analyzed by means of SDS-PAGE and Matrix-assisted laser desorption ionization time-of-flight mass spectrometry (MALDI-TOFMS) in an Applied Biosystems 4700 Proteomics Analyzer.

The N-terminal amino acid sequence of cordymin was analyzed by means of Automated Edman Degradation using a Hewlett Packard 1000A protein sequencer equipped with an HPLC system.

### 2.2. Experimental Design

Thirty Sprague-Dawley male rats were divided into 5 groups (*n* = 10) at random. The rats in the diabetic and cordymin treatment groups were fasted for 10 h and intraperitoneally injected with 75 mg/kg of alloxan to induce diabetes; the remaining 10 control rats were also fasted for 10 h and injected with 0.9% saline. Forty-eight hours after the injection, blood glucose was >16 mmol/L and urine glucose was >+ indicating that diabetes was successfully induced in the rats. The rats in the cordymin treatment group were intraperitoneally injected with cordymin (20, 50, and 100 mg/kg/day) for 5 weeks, and the other 2 groups of rats were intraperitoneally injected with saline (6 mL/kg/day). The body weights of the animals were recorded weekly during the experimental period.

At the end of the experimental period, the animals were fasted overnight (18 h) and then sacrificed by decapitation, and the blood was collected to be centrifuged at 3000 rpm for 20 min, and the clear serum was separated for biochemical analysis. The pancreas was dissected out and placed in 10% buffered formalin, and the liver was dissected out for the measurement of total antioxidant status (TAOS). Femurs were dissected and filled in physiological saline and stored at −20°C for measurement of total bone mineral content (BMC) and bone mineral density (BMD) by Dual-energy X-ray absorptiometry.

### 2.3. Biochemical Methodology

The blood glucose was analyzed with a Glucometer-4 (Bayer) and HbA1c with the HbA1c Apparatus (Variant II, Bio-Rad Laboratories). Serum insulin level was determined with an enzyme-linked immunosorbant assay (ELISA) kit (Nanjing Jiancheng Bioengineering Co. Ltd., China). Serum alkaline phosphatase (ALP) and tartrate resistant acid phosphatase (TRAP) activity were determined by nitrophenol-based method as described by Bessy et al. [12] and Godkar [13], respectively.

### 2.4. Estimation of the Damaged Pancreatic *β* Cells

The pancreatic tissues were embedded in paraffin blocks after formalin fixation. Paraffin sections were cut at 4 *μ*m thickness and were deparaffinized in xylene twice for 5 min and then were rehydrated with the graded ethanol. The sections were examined after hematoxylin and eosin (H&E) staining. 

### 2.5. Estimation of the Total Antioxidant Activity

The total antioxidant status (TAOS) of hepatic tissue was determined by the way introduced by Laight et al. [14]. The increase in absorbance at 405 nm was measured by using a microplate reader (Shanghai Xunda Medical Technology Inc., China).

### 2.6. Estimation of Bone Mineral Content (BMC) and Bone Mineral Density (BMD)

The left femur and L-4 vertebra were mineralized at the temperature of 620°C for 48 h and weighed. The mineralized bones were dissolved in 6 M HCl, and then calcium content in the bone mineral content was assayed by a colorimetric method. BMD was calculated by BMC of the measured area.

## 3. Results

### 3.1. Effect of Cordymin on the Body Weights of the Diabetic Osteopenic Rats

**Figure 2:**
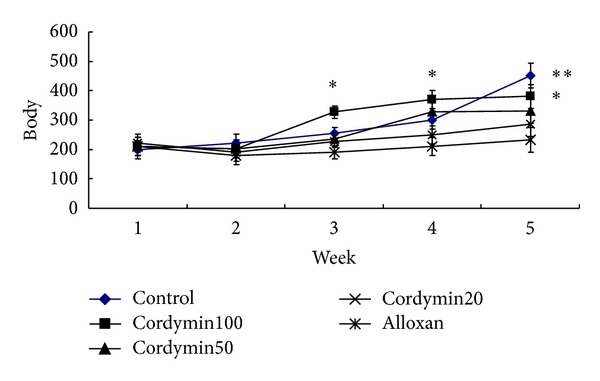
Effects of cordymin on the weekly average body weights. Values are means ± SEM, *n* = 10. *Different from alloxan group *P* < 0.05; **different from alloxan group, *P* < 0.01.

The body weights of the hyperglycemic rats induced by alloxan are presented in Figure 2. The initial body weights of the rats were similar between groups. The body weights of the alloxan-induced diabetic rats were significantly lower than that of the control rats. Contrasted with the diabetic group, the body weights of rats in cordymin-treated group were increased gradually 21 days later (*P* < 0.05, *P* < 0.01).

### 3.2. Effect of Cordymin on the Blood Glucose and HbA1c Levels of the Diabetic Osteopenic Rats

The results of blood glucose from hyperglycemic rats induced by alloxan are presented in Table 1. The serum glucose levels of the saline-treated diabetic rats were significantly higher than that of other rats (*P* < 0.05). Treatment with cordymin at 50 mg/kg and 100 mg/kg significantly lowered the serum glucose level in diabetic rats (*P* < 0.05). Meanwhile, cordymin could decrease the concentration of HbA1c in plasma of alloxan-induced hyperglycemic group 5 weeks later (*P* < 0.01),as shown in Table 1.

### 3.3. Effect of Cordymin on Serum Insulin of the Diabetic Osteopenic Rats

As shown in Table 2, the levels of serum insulin elevated after administration of cordymin100 (*P* < 0.05). However, the same results did not occur in other groups throughout the total duration of the study.

### 3.4. Effect of Cordymin on Plasma Enzyme of the Diabetic Osteopenic Rats

In the present study, significant increase in ALP and TRAP levels, two bone formation markers, was observed in alloxan-induced hyperglycemic group (Table 3). On the contrary, cordymin at 100 mg/kg significantly decreased ALP and TRAP levels (*P* < 0.01) in diabetic rats, while cordymin at 50 mg/kg and 20 mg/kg had little influence on the two bone formation markers in diabetic rats.

### 3.5. Effect of Cordymin on the Damaged Pancreatic *β* Cells

**Figure 3:**
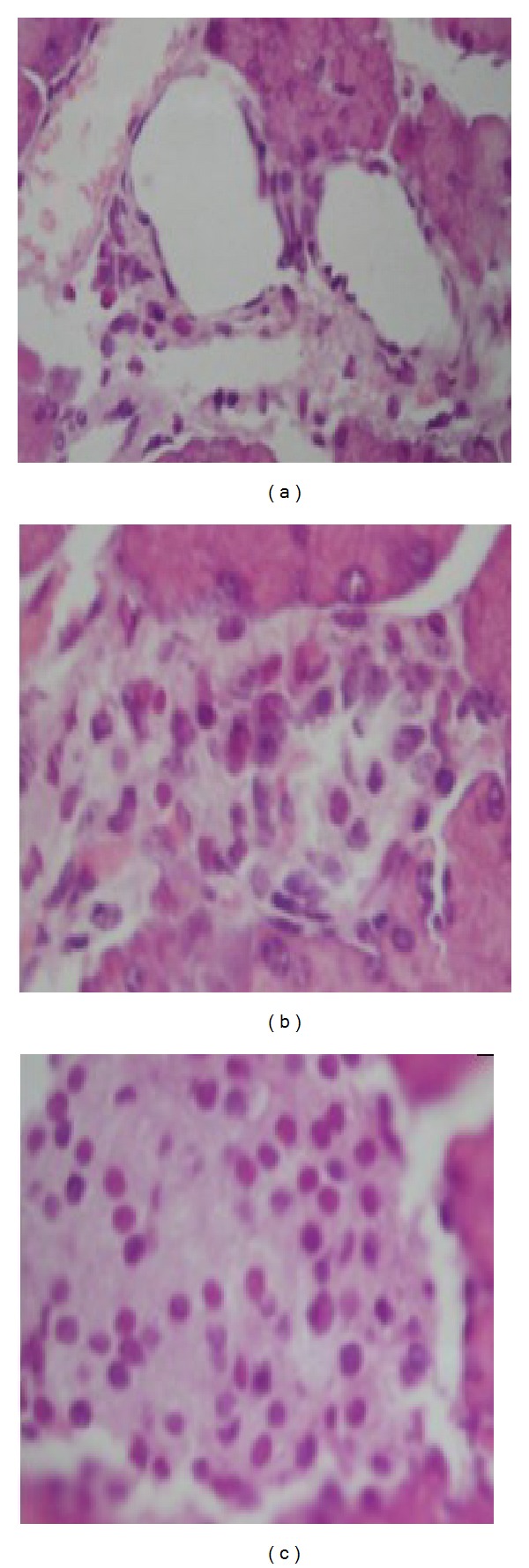
Islet cell death and replication represented by hematoxylin-eosin. The islet cells of diabetic rats of alloxan treatment (a) showed extensive cell lysis, representing loss of plasma membrane with condensed nuclei and dissolved cytoplasm in wide intercellular spaces. In contrast, the islet cell of cordymin100-fed rat (b) was partly recovered. The islet cell of control rat was (c).

Histopathological evaluation revealed severe *β* cells death in the diabetic rats (Figure 3(a)). In contrast, such loss of cells was not seen in the islet cells of the control rats (Figure 3(b)). The *β* cells of the rats fed with cordymin100 were partially recovered (Figure 3(c)).

### 3.6. Effect of Cordymin on Total Antioxidant Activity of the Diabetic Osteopenic Rats

**Figure 4:**
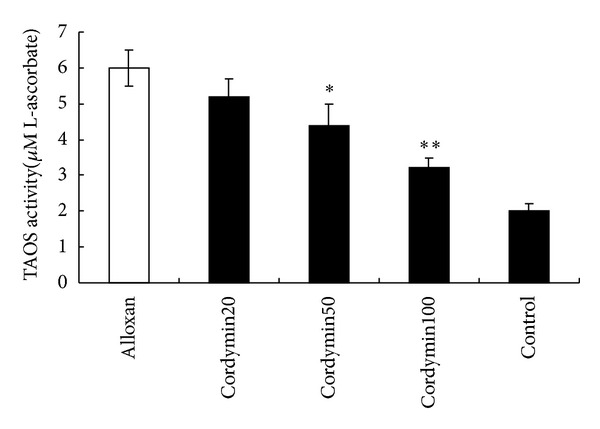
Effect of cordymin on total antioxidant. Activity values are shown as means ± SEM. *P < 0.05 versus alloxan group; **P < 0.01 versus alloxan group.

We measured TAOS activity as an indirect indication of the formation of O_2_^−^ and other oxidant species. This index was increased in the diabetic groups induced by alloxan in comparison with the control group. In contrast, the TAOS activities of both the cordymin100 and cordymin50 groups were lower than those of diabetic group (*P* < 0.05 and *P* < 0.01) (Figure 4).

### 3.7. Effect of Cordymin on BMC and BMD

**Figure 5:**
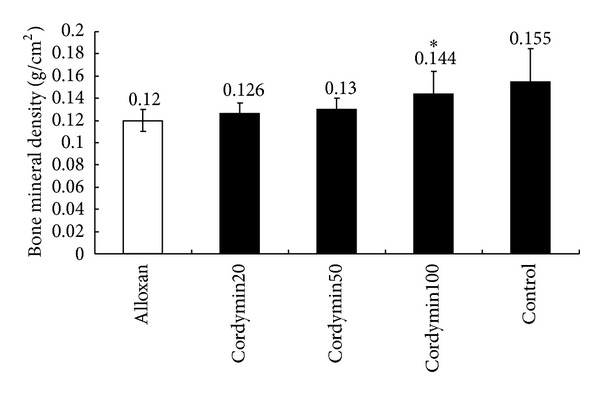
Effect of cordymin on bone mineral density (BMD). Values are shown as means ± SEM. *P < 0.05 versus alloxan group.

The BMC in the examined bones of the diabetic groups was significantly reduced compared to the results obtained for the controls. The administration of cordymin100 and cordymin50 to the diabetic animals increased significantly the BMC in the examined bones when compared to the diabetic group (Table 4). Meanwhile, cordymin at 100 mg/kg significantly increased the femur BMD in diabetic rats (*P* < 0.05) (Figure 5).

## 4. Discussion

The wide spread chronic disorder of diabetes mellitus adversely affects multiple organ systems including bones. One of the serious skeletal complications in bones is osteoporotic fractures due to weakened bone strength. The relationship between diabetes and osteoporosis is widely reported [15–17]. Rat models of diabetic osteopenia have contributed significantly to the pathophysiologic understanding of these clinical challenges with regard to bone turnover, bone regeneration, and pharmacologic therapies [18, 19]. However, the animal model of diabetic osteopenia was usually induced with streptozotocin (STZ), and fewer models were induced with an intravenous dose of alloxan. In the present study, we investigated the effects of cordymin on diabetic osteoporosis using the alloxan-induced diabetic rat model.

Alloxan diabetes has been commonly utilized as an animal model of insulin-dependent diabetes mellitus (IDDM). Alloxan produces selective cytotoxicity in pancreatic *β* cells through the generation of reactive oxygen species resulting in reduced synthesis and release of insulin [20]. In the present study, we examined the antidiabetogenic effect of cordymin by monitoring the *β* cells number, and islet mass was assessed by morphometry on immunostained tissue sections. The *β* cells death and alteration of islet cell population were prominent in the alloxan-induced diabetic rats (Figure 1(a)). In addition, alloxan-induced type 1 diabetes reduced bone quality. Alloxan-treated rats showed a decrease in femoral BMC and BMD compared to normal control rats (Table 4 and Figure 5). It demonstrated the significant association between diabetes and osteoporosis.

The present study was performed to investigate the effectiveness of cordymin on diabetic osteoporosis. Cordymin-treated rats, indeed, exhibited the recovery of *β* cells. It is consistent with the results of cordymin treatment on insulin levels. At the same time, BMC and BMD in diabetic rats treated with cordymin increased when compared to the diabetic rats. Therefore, the beneficial effect of cordymin on pancreatic tissue and its capability of improving serum insulin level might be, at least in part, responsible for the protective effect of cordymin on diabetic osteopenia in the present study.

Hyperglycemia is able to trigger increased oxidative stress, which has been considered to be involved in the pathogenesis of diabetic bone disorders [21]. Cordymin could lower the blood glucose and HbA1c levels, which have been shown to trigger decreased TAOS in diabetic rats. Therefore, the effect of cordymin on diabetic osteopenia in the present study might also be realized indirectly through lowering the concentration of serum glucose, which subsequently triggered a lower extent of oxidative stress in diabetic rats.

Serum alkaline phosphatase (ALP) is a noncollagenous protein secreted by osteoblast, which is essential for bone mineralization [22]. Increased ALP level in serum has been observed in conditions such as rapid bone loss [23] and fracture risk [24, 25]. Tartrate resistant acid phosphatase (TRAP) is secreted by osteoclasts during bone resorption, and serum TRAP activity correlates with resorptive activity in disorders of bone metabolism. In the present study, cordymin significantly decreased ALP and TRAP levels commonly using bone remodeling markers. It suggested that the potency of cordymin is due to decrease of ALP activity and TRAP activity in diabetic rats.

In conclusion, the present study showed for the first time that administration of cordymin has significant effects in rat model of diabetic osteopenia, including dose-dependently restoring the circulating blood glucose, HbA1c, ALP, TRAP, and insulin levels. The beneficial effect of cordymin on diabetic osteopenia might be directly through lowering ALP and TRAP activity and indirectly through recovery of *β* cells and lowering the concentration of serum glucose, which subsequently triggered a lower extent of oxidative stress in diabetic rats. 

## Figures and Tables

**Table 1:** Effect of cordymin on blood glucose and HbA1c levels in alloxan-diabetic osteopenic rats.

Different groups	Blood glucose (mmol/L)	Results of HbA1c (%)
Alloxan-treated	21.0 ± 2.0	10.8 ± 0.20
Cordymin100-treated	13.2 ± 3.0*	8.0 ± 0.30*
Cordymin50-treated	19.2 ± 3.2*	10.0 ± 0.25
Cordymin20-treated	18.8 ± 2.5	9.8 ± 0.30
Control group	6.0 ± 1.5	4.8 ± 0.21

Values are shown as means ± SEM, *n* = 10. *P < 0.05 versus alloxan group.

**Table 2:** Effect of cordymin on serum insulin level in alloxan-diabetic osteopenic rats.

Different groups	Serum insulin (*μ*U/mL)
Alloxan-treated	4.4 ± 1.1
Cordymin100-treated	8.4 ± 0.2*
Cordymin50-treated	6.1 ± 1.1
Cordymin20-treated	5.5 ± 1.0
Control group	8.5 ± 1.2

Values are shown as means ± SEM, *n* = 10. *P < 0.05 versus alloxan group.

**Table 3:** Effects of cordymin on plasma enzymes in alloxan-diabetic osteopenic rats.

Different groups	TRAP level (uM)	ALP level (mM)
Alloxan-treated	0.83 ± 0.11	7.20 ± 0.10
Cordymin100-treated	0.46 ± 0.03*	4.00 ± 0.03*
Cordymin50-treated	0.50 ± 0.01	5.90 ± 0.05
Cordymin20-treated	0.70 ± 0.05	6.30 ± 0.05
Control group	0.20 ± 0.11*	3.25 ± 0.12

Values are shown as means ± SEM, *n* = 10. *P < 0.05 versus alloxan group.

**Table 4:** Effects of cordymin on bone mineral content.

Different groups	Total bone mineral content (mg)	Calcium content (mg/g)
Control group	309.05 ± 8.16^a^	389.65 ± 10.22^a^
Alloxan-treated	263.26 ± 5.63^b^	326.25 ± 9.66^b^
Cordymin100-treated	316.11 ± 6.20^a^	443.21 ± 12.23^a^
Cordymin50-treated	316.11 ± 6.20^a^	443.21 ± 12.23^a^
Cordymin20-treated	316.11 ± 6.20^a^	443.21 ± 12.23^a^

The different letters in the same column indicate a statistical difference (*P* < 0.05).
